# Rhus Glabrum, for Ptyalism

**Published:** 1829

**Authors:** 


32. *Rhus Glabrum, a remedy for Ptyalism.* Dr. Fahnestock, Am. Jour. for Nov. 1829, has recommended the bark of this shrub, the common sumach of our woods and fields, as an excellent remedy for salivation and mercurial ulceration of the mouth.

"An infusion, says he, of the inner bark of the root of the Rhus glabrum is a very mild mucilaginous refrigerant, and moderately astringent, very cooling and soothing to the irritated surface of the mouth and throat, and can be applied in any stage of disease and at any age. It acts by allaying irritation and obstructing excitement, sheathing the delicate surfaces, and healing abrasions."

Dr. F. has given the notes of some severe cases of mercurial disease in the mouth, which yielded, without delay, to this infusion.

